# Evaluating Patterns and Factors Related to Sleep Disturbances in Prostate Cancer Patients

**DOI:** 10.3390/healthcare10050832

**Published:** 2022-04-30

**Authors:** Shalini Mondal, Steve Edwards, Erik Wibowo, Hashim Ahmed, Richard J. Wassersug, Jason Ellis, Maximus Isaac, Dagmara Dimitriou, Stephen Mangar

**Affiliations:** 1 Sleep Education and Research Laboratory (SERL), UCL Institute of Education, London WC1H 0AA, UK; shalini.mondal@kcl.ac.uk (S.M.); 2001084@buckingham.ac.uk (M.I.); 2 Clinical Trials Department, Imperial College Healthcare NHS Trust, London W6 8RF, UK; edwards@sketters.co.uk; 3 Department of Anatomy, University of Otago, Dunedin 9016, New Zealand; erik.wibowo@otago.ac.nz; 4 Department of Urology, Imperial College Healthcare NHS Trust, Charing Cross Hospital, London W6 8RF, UK; hashim.ahmed@nhs.net; 5 Department of Cellular and Physiological Sciences, Faculty of Medicine, University of British Columbia, Vancouver, BC V6T 1Z3, Canada; richard.wassersug@ubc.ca; 6 Northumbria Sleep Research Laboratory, Faculty of Health and Life Sciences, Northumbria University, 134/408 Northumberland Building, Newcastle upon Tyne NE1 8ST, UK; jason.ellis@northumbria.ac.uk; 7 Department of Clinical Oncology, Imperial College Healthcare NHS Trust, Charing Cross Hospital, London W6 8RF, UK

**Keywords:** sleep, insomnia, prostate cancer, actigraphy, sleep fragmentation

## Abstract

Prostate cancer patients may experience disturbed sleep as a result of their diagnosis or treatment. This study sought to evaluate disturbed sleep and excessive daytime sleepiness in newly diagnosed patients and those receiving androgen deprivation therapy (ADT). This study was conducted with 74 patients. Subjective data using the Pittsburgh Sleep Quality Index (PSQI) and Epworth Sleepiness Scale (ESS) and actigraphy data on ADT/ADT-naïve patients were collected. The prevalence of poor sleep quality, determined from PSQI and ESS scores, was 50% and 16.7% respectively. Those on ADT (n = 20) had poorer sleep quality as determined by significantly higher PSQI scores (70 vs. 40% scoring > 5) and were more likely to have poor sleep quality, sleep latency, and sleep efficiency than ADT-naïve patients (n = 40). Actigraphy data showed that ADT patients slept significantly longer (7.7 vs. 6.8 h), experienced a higher Fragmentation Index (48.3 vs. 37.4%), and had longer daytime nap duration (64.1 vs. 45.2 min) than ADT-naïve patients. The use of objective measures such as actigraphy in the clinical arena is recommended and may be used as a valuable tool for research into sleep assessment in prostate cancer patients.

## 1. Introduction

Individuals with cancer are disproportionately affected by sleep disturbances compared with the general population [1], with a prevalence ranging from approximately 30% to 75% in newly diagnosed or recently treated cancer patients [2]. In addition, poor sleep can be detrimental for overall health, being a cause and consequence of chronic pain [3] and a risk factor for cognitive decline and dementia [4,5].

Prostate cancer is one example where treatment, in addition to the diagnosis itself, can affect sleep quality and impact overall quality of life (QoL). Sleep disturbances may arise following radical prostatectomy or radiation therapy [6]. Androgen Deprivation Therapy (ADT), which is widely employed in both metastatic and locally advanced prostate cancer, is highly associated with poor sleep and this is likely to be related to nocturnal hot flashes and/or the loss of androgen [7,8,9]. In practice, however, sleep issues are rarely assessed, diagnosed, and treated, as compared with the more recognizable longer-term side effects such as osteoporosis, cardiovascular and diabetic complications associated with ADT. Moreover, a distinction between sleep-related reduced daytime functioning fatigue and cancer-related fatigue is yet to be clinically characterized. As such, it remains unknown whether ADT can affect sleep quality in addition to daytime fatigue, or indeed whether the two are linked.

It is important to distinguish sleep-related fatigue from cancer-related fatigue (CRF), where patients still feel tired despite a relatively normal amount of night-time sleep [10], as the two conditions are likely to require different management strategies. Nevertheless, CRF can occur in parallel with, and may be reciprocally related to, poor sleep. As such, the effective management of sleep and sleep disorders may reduce CRF and vice versa.

Sleep quality and sleepiness are generally assessed subjectively by patients using questionnaires such as the Pittsburgh Sleep Quality Index, and the Epworth Sleepiness Scale [11]. A gold standard measure of sleep is via the use of polysomnography [12]; however, its usage within this clinical setting is expensive and difficult to implement in everyday clinical practice. Actigraphy has been introduced into the clinical arena as a more convenient portable way of monitoring sleep based on patient movement, with a sensor that can be worn in wristwatch form. However, most studies on prostate cancer patients assessed sleep outcomes using questionnaires, and rarely used actigraphy.

There remains a need to investigate the prevalence of sleep problems and detail the sleep patterns and daytime sleepiness in patients with prostate cancer using a combination of subjective (i.e., sleep questionnaires) and objective (i.e., actigraphy) methods. Given the limited research on the trajectory of sleep in patients with prostate cancer, an additional aim is to explore sleep patterns between patients receiving ADT and those who were ADT naïve.

## 2. Materials and Methods

### 2.1. Participants

Data were prospectively collected from 74 patients as part of a larger longitudinal study examining sleep patterns in prostate cancer patients, conducted at Imperial College Healthcare NHS Trust. Here we sought to confirm the feasibility of using wrist actigraphy along with questionnaire data in the clinical setting. We also aimed to collect initial data on trends in sleep prevalence for the purposes of hypothesis generation, power calculation and refinement of the methodology. 

Eligible patients were required to have histologically proven prostate cancer diagnosis, either newly diagnosed or already on ADT. Exclusion criteria included: diagnosis of sleep apnea, any psychiatric condition that interferes with study compliance, dementia, and Parkinson’s disease. For the purposes of this analysis, men who were receiving systemic chemotherapy or newer androgen blockers, such as Abiraterone or Enzalutamide, were also excluded.

Rather than designate a specific number of participants for accrual, we chose a time interval of 9 months for study recruitment, to assess the feasibility of collecting objective data using wrist actigraphy, and questionnaire data in a clinical out-patient setting. This was felt to be a reasonable time period upon which an interim assessment could be performed. It also allowed us to interrogate the data and help refine our methodologies, while collecting an adequate sample size to power the next phase of the study.

### 2.2. Procedure 

During the hospital visit, each participant was provided with a full description of the study and details about the actigraphy. Once informed consent was obtained, participants completed a set of questionnaires and were given an actigraphy watch to wear on the non-dominant wrist for a period of one week. The questionnaires were self-completed or completed with the help of the researcher if participants had poor vision and/or difficulties with writing. The analysis for this study was limited to datasets where both actigraphy and questionnaire data were available and only included patients who were ADT naïve or those receiving luteinizing hormone-releasing agonists (LHRHa). In this paper, ADT is restricted specifically to those on LHRHa.

### 2.3. Measures 

#### 2.3.1. Background Data

Sociodemographic and medical information, including height, weight, Body Mass Index (BMI), age at diagnosis, ethnicity, clinical stage at data collection, medical co-morbidities (e.g., diabetes mellitus, ischemic heart disease, and hypertension), nocturia, smoking history and alcohol consumption were obtained at baseline from clinician records at the time of consultation. 

#### 2.3.2. Subjective Sleep Measures

Sleep Quality was measured using the Pittsburgh Sleep Quality Index (PSQI), a retrospective self-report questionnaire measuring sleep quality over the previous month, using 19 items [13]. The self-rated items vary from 0 (not during the past month) to 3 (three or more times during the past month). Items included in the PSQI measure sleep disturbances across seven dimensions: (1) subjective sleep quality, (2) sleep latency, (3) sleep duration, (4) habitual sleep efficiency, (5) sleep disturbances, (6) use of sleep medication and (7) daytime dysfunction. These subscales are summed to produce a global PSQI score which ranges from 0 to 21. Higher global scores on the PSQI indicate worse sleep quality, with a cut off score of >5 indicating a clinically relevant sleep disturbance. The clinical properties of PSQI demonstrate its utility in both clinical practice and research [11].

Daytime sleepiness was assessed using the Epworth Sleepiness Scale (ESS). The ESS is an 8-item self-administered questionnaire, designed to evaluate overall daytime sleepiness [14]. Participants completing the ESS are instructed to rate their usual chance of dozing or falling asleep while engaged in different activities (where 0 indicates that they would never doze and 3 suggesting a high chance of dozing). Total ESS scores range from 0 to 24, and a score >10 suggests clinically significant daytime sleepiness [11]. 

#### 2.3.3. Objective Sleep Measures

##### Actigraphy

The MotionWatch8, manufactured by CamNtech, is a CE marked Class 1 medical device with FDA approval (K132764) with an internal miniature accelerometer. The actigraph was used to collect movement samples in 30 s epochs. Extracted data of interest in this study included: Total Sleep Time (TST) i.e., actual time asleep, categorized by wake/sleep categorization. Fragmentation Index (FI) i.e., degree of movement during the night, an indication of sleep quality. Sleep Efficiency (SE) i.e., TST expressed as a percentage of time in bed. Wake-Bout Frequency i.e., the number of contiguous wake sections in the epoch-by-epoch wake/sleep categorization. Wake-Bout duration i.e., the average length of each of the wake bouts in minutes. Daytime Napping Duration (min), and Daytime Napping Frequency [15]. Data were analyzed using the CamNtech Motionware software on a computer. 

### 2.4. Statistical Analysis

Actigraphy data were downloaded using CamNtech Motionware software. SPSS version 26 (IBM Corporation, Armonk, NY, USA) was used to analyze the data. PSQI and ESS scores were used as outcome measures of sleep quality and daytime sleepiness, respectively. Results are given as Mean ± SD unless otherwise stated. In cases where the data were skewed, the median and Inter Quartile Range (IQR) are also reported. Appropriate parametric or non-parametric tests were then chosen to compare variables between patient groups and to explore associations between variables. 

Continuous data (demographic and sleep–wake data) were compared between groups using either a *t*-test or Mann–Whitney U test depending on normality. Categorical variables were compared between groups using the chi-squared test. Spearman’s correlation analysis was used to determine any association between the objective and subjective sleep data. Multiple regression analyses were performed to determine the association of ADT treatment and actigraphic variables, after controlling for age, ethnicity, WHO performance score and T cancer staging. A *p* value of <0.05 was considered significant.

## 3. Results 

### 3.1. Missing Data

An actigraphy watch failure rate of 14.8% was observed. Failure was defined as the inability to retrieve actigraphy data, either due to a technical failure (i.e., battery leakage, water seepage) or poor patient compliance (i.e., lack of understanding regarding the need to wear the watch continuously). The questionnaire completion rate was 97.2%. The missing data were due to one patient declining to complete a questionnaire and another questionnaire being misplaced in the post. 

### 3.2. Demographic

**Figure 1:**
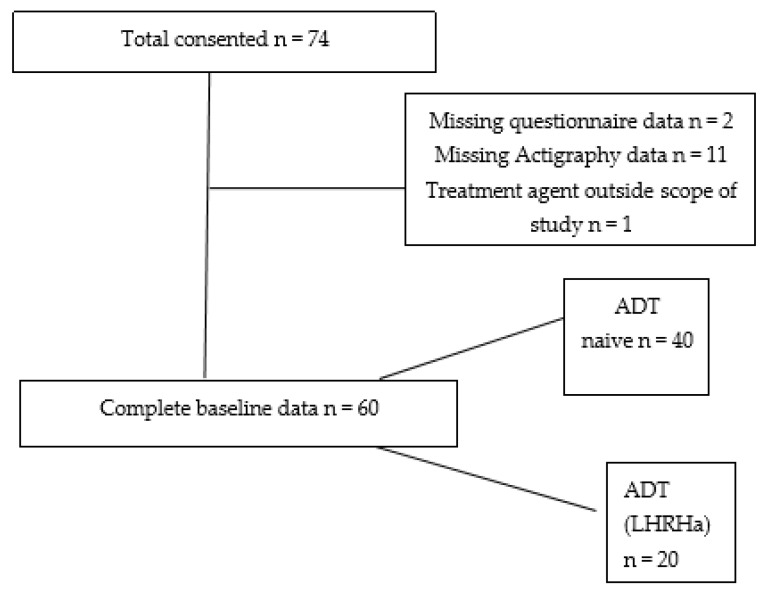
Consort diagram of patient data collection and assessment.

Of the 74 patients recruited into the study, completed actigraphy and questionnaire data sets were available for 61 patients (82.4%). One patient had to be excluded as he was on a hormonal agent outside the scope of this study, leading to the final assessment being conducted on 60 patients. From this final cohort of 60 patients, 20 were receiving LHRHa injections (given as a three-monthly depot injection), and 40 were ADT naïve, i.e., were new patients to the clinic who were yet to start treatment or were on an active surveillance program. This is summarized in Figure 1.

The baseline characteristics for the cohort of 60 patients are summarized in Table 1.

Overall, the ADT naïve group were significantly younger, diagnosed at an earlier age, with a lower-presenting PSA than those receiving ADT in the form of LHRHa. Patients on ADT were more likely to have poorly differentiated disease, with more nodal and distant metastases compared with ADT naïve patients. 

### 3.3. Prevalence of Sleep Problems

Both questionnaire and actigraphic prevalence data are summarized in Table 2.

From the questionnaires, the prevalence of poor sleep quality irrespective of treatment (as defined by PSQI score > 5) and excessive daytime sleepiness (as defined by ESS score > 10) was 50% and 16.7%, respectively. The proportion of patients with an ESS of >10 did not differ by treatment, but ADT-naïve patients were less likely to have a PSQI score > 5 compared with patients on ADT (*p* < 0.05). 

The actigraphy data shows that 43.4% of the study population had a TST of less than 7 h, 38.4% had a poor SE (where an SE < 80% is associated with increased mortality risk in the elderly [13]), 58.4% experienced a high FI (where FI ≥ 40% is associated with poor sleep quality [14]), whilst 43.4% experienced an excessive daytime napping duration, based on a pre-defined cutoff of 60 min [15].

### 3.4. Correlation between Actigraphy and Questionnaire Data

Significant correlations were observed between the ESS, and actigraphic daytime napping duration (rho = 0.34, *p* < 0.01), as well as ESS and actigraphic daytime napping frequency (r = 0.31, *p* < 0.05). Moreover, Global PSQI scores correlated with the actigraphic wake-bout frequency (r = 0.26, *p* < 0.05). The sleep latency sub-component of the PSQI was also correlated with the actigraphic fragmentation index (r = 0.30, *p* < 0.05) and actigraphic wake-bout frequency (r = 0.46, *p* < 0.0001). Lastly, the sleep disturbance score was correlated with actigraphic sleep latency (r = 0.36, *p* < 0.01).

### 3.5. Differences in Sleep Outcomes According to Treatment

#### 3.5.1. Sleep–Wake Parameters from Questionnaire Data

Patients on ADT were noted to have a significantly higher global PSQI (*U* = 280.0, *p* < 0.001) but not significantly different ESS scores (*U* = 311.5, *p* > 0.05) compared with ADT-naïve patients. They were also more likely to have a higher score for subjective sleep quality (χ^2^(1) = 8.3, *p* < 0.05), sleep latency (χ^2^(1) = 14.4, *p* < 0.01) and habitual SE (χ^2^(1) = 12.6, *p* < 0.01) components of the PSQI than the ADT-naïve patients. This is summarized in Table 3. 

#### 3.5.2. Sleep–Wake Parameters from Actigraphic Data

Data from the actigraphy indicated that patients on ADT had significantly longer sleep time (*t* = −2.5, *p* < 0.05), more frequent awakening (*U* = 242.0, *p* < 0.05), higher FI (*U* = 216.5, *p* < 0.01), longer nap duration (*U* = 262.5, *p* < 0.05), and more frequent naps (*U* = 243.0, *p* < 0.05) than the ADT-naïve patients. Their SE and wake-bout duration were similar regardless of treatment type. These results are summarized in Table 4.

Table 5 shows that after adjusting for age, ethnicity, WHO Performance Score and cancer staging, ADT treatment is associated with increased wake-bout frequency (β = 0.385, *p* = 0.021), but not for other actigraphic measures.

## 4. Discussion

The aim of this observational/feasibility study was to discern trends in the prevalence and sleep patterns in prostate cancer patients using a combination of subjective (questionnaires) and objective (actigraphy) data. Whilst polysomnography is considered the gold standard to objectively measuring sleep, it requires a dedicated sleep laboratory and is not conducive for use in a clinic setting with a large cohort of patients. In this study, the combined use of wrist actigraphy and completing questionnaires was satisfactory with 85% and 98% compliance rates, respectively. 

Most of the actigraphy failures were due to technical problems with setting up the device, and these were generally overcome after the first 10 patients. Whilst sleep diaries were encouraged, uptake was poor, and a decision was taken to discontinue their use after negative feedback from the first five patients. The premise for including actigraphy data was to interrogate the questionnaire data knowing that, if a specific sleep disturbance or indicator of daytime sleepiness (i.e., napping) could be identified, then those patients could potentially be directed to specific therapeutic interventions.

Whilst there are at least two main prospective studies that have considered the impact of ADT on sleep problems [16,17], and a further study investigating ADT with radiation therapy [9], none has used actigraphy in their clinical assessment. The current study showed that sleep disturbances were higher for the cohort of patients on ADT compared with those who were ADT naïve (70% vs. 40%). This suggests that hormonal treatment by itself may impact on a patient’s subjective quality of sleep. It may also be confounded by the older age and more advanced disease stage of the ADT LHRHa cohort, as highlighted in the regression analysis of the actigraphy parameters. 

The current study also provides further evidence on excessive daytime napping, which was identified via the ESS scale in 16.7% of the whole cohort. The prevalence of sleep disturbances, as assessed using PSQI, correlated well with the prevalence rates of selected actigraphic parameters evaluating sleep quality. However, the prevalence rate of excessive daytime sleepiness, as assessed by the ESS, was significantly lower than the prevalence of the objective daytime napping duration recorded by actigraphy. Indeed, an ESS score of 10–15 suggests significant daytime sleepiness requiring medical attention in certain situations. The actigraphy data suggest that some patients may nap during the daytime under a variety of situations that are different from those captured in the ESS. 

Of the seven parameters assessed by actigraphy, all except wake-bout frequency, wake-bout duration and daytime napping frequency have published normative ranges [18,19,20]. As such, based on the cohort of 60 patients, analysis of actigraphic parameters showed that irrespective of treatment, 43.4% of the study population had reduced Total Sleep Time (TST), which is less than the recommended 7 h. Some 38.4% had poor quality sleep, as indicated by an SE of <80%.

When considering whether questionnaire or actigraphy data better define the impact of hormonal treatment on prostate cancer patients, several outcomes stand out. For example, subjective sleep quality was significantly higher in terms of median Global PSQI in the ADT group than the treatment-naïve group (7 vs. 4, *p* < 0.01). However, actigraphy showed a longer sleep duration for patients on this treatment by 50 min (7.7 ± 1.4 h compared with 6.8 ± 1.3 h, *p* < 0.05). This difference is not reflected in the questionnaire data, as the latter asks patients specifically about sleep time. This points to a substantial difference between objective and subjective assessments.

Similarly, the actigraphy parameters of Wake-Bout frequency and FI show a significant difference between groups, with a more disturbed sleep pattern for those on ADT (39.2 vs. 46.2, *p* < 0.05, and 48.3% vs. 37.4% *p* < 0.01). However, there was no difference in sleep efficiency between the two groups. These findings were supported by regression analysis.

Whilst both daytime napping duration and frequency were significantly greater for those receiving ADT, this difference was not seen in the ESS scores or the regression analysis. It is noteworthy, though, that patients with a higher fragmentation index have a greater duration of daytime sleepiness [21]. 

Taken together, both questionnaire and actigraphy data suggest that patients on ADT have a more fragmented pattern of sleep. They probably compensate for this by sleeping for a longer duration at night and during the day. These data are consistent with LHRHa having a negative impact on sleep patterns for prostate cancer patients, which is revealed by our relatively small cohort of patients. Considering the actigraphy parameters collectively, the data do not seem to be at odds with the questionnaire data. Rather they help define the sleep problems more explicitly.

It is interesting to note that whilst those on ADT had a more fragmented sleep pattern and were sleeping for longer, this was not reflected by a commensurate reduction in the SE, either with the questionnaires or the actigraphy data. This is discordant to what would be expected in terms of SE decreasing with increasing age, given that the ADT treatment cohort was generally older [22]. This may relate to the way in which SE is determined. Sleep duration and quality are known to be impacted by environmental factors such as such as reading, texting, conversing with a partner, watching television both prior to initiating and after final awakening; this makes SE inherently subjective. In contrast, the actigraphic data determine SE by recording total sleep/wake onset duration from actigraphic parameters, which reflect total sleep time compared to the amount of time awake over the sleep period. By examining the actigraphic parameter of duration of daytime napping in the 10 patients with an abnormal ESS, 50% demonstrated more than 60 min of daytime napping on actigraphy. Such daytime napping of that duration is associated with poor mental and physical health [19].

A significant limitation of this predominantly hypothesis-generating feasibility study is the notable age and disease status difference between the ADT and ADT-naïve groups, and the discrepancy in number between the groups. Those receiving ADT were older and had a greater burden of disease with more aggressive tumors. Admittedly, these differences are expected when men on ADT are compared with men on PSA surveillance. Furthermore, the ADT-naïve cohort tended to be fitter, with fewer co-morbidities, consumed less alcohol and were less likely to smoke; however, this was not statistically significant. 

Nevertheless, regression analysis—after controlling for age, cancer staging, performance score and ethnicity—showed the only difference to be that wake-bout frequency was significantly influenced by these other variables. All these potentially confounding factors should be borne in mind when interpreting the findings of this feasibility study. A larger, adequately powered study is needed to ratify our findings. In future studies, we aim to collect both questionnaire and actigraphy data at serial time points where each participant can act as his own control and thus mitigate against such confounding variables.

The notable discrepancy in numbers between the ADT (n = 20) and the ADT naïve (n = 40) is related to the fact that most of the patients attending the prostate clinic were either newly diagnosed and needed prompt ADT treatment or were on PSA surveillance. These numbers will be expanded with a greater balance in stratification in the second phase of this study.

As acknowledged previously, insomnia and CRF are different entities, but are frequently associated. This study has not assessed the impact of CRF, mainly because patients were not compliant with sleep diaries. Literature suggests that actigraphy can be used to assess CRF by tracking such parameters as Wake-After-Sleep Onset (WASO) and Sleep-Onset Latency (SOL). The latter cannot be fully evaluated without the use of sleep diaries to define the SPT (Sleep Period Time) required for processing the actigraphy data [23]. 

Only one form of hormonal treatment (i.e., LHRH analogue injections) was assessed in this study. It would be interesting to explore the potential differential impact of other hormonal therapies, including newer androgen synthesis inhibitors and androgen receptor-targeting agents. 

Finally, the impact of hot flashes and sleep disturbance was not assessed in this study. It is known that a significant proportion of patients receiving ADT experience insomnia associated night sweats [24]. It may well be that using a hot flash severity scale may help interpret the actigraphy data [25].

## 5. Conclusions

The study highlights the feasibility of using actigraphy in this clinical setting and allows for a better understanding of the relationship between sleep questionnaires and actigraphy. The questionnaires inform “if” the patients are sleeping poorly, whilst the actigraphy reveals “how” they are sleeping poorly. For prostate cancer patients, actigraphy provides new insights into the nature of sleep disturbances in ADT patients who may then be amenable to therapeutic interventions, thereby improving their lives. 

In addition, using actigraphy to objectively assess sleep quality can be applied in clinical trials to determine the impact that new oncological treatments have on sleep quality and, hence, quality of life.

## Figures and Tables

**Table 1:** Demographic and clinical characteristics of participants who were ADT naïve or on ADT with an LHRHa drug. Data are presented as either mean ± standard deviation or median (interquartile range), depending on data normality.

Total Participants (n = 60)	ADT Naïve (n = 40)	ADT (LHRHa) (n = 20)
Characteristic	Mean ± S.D. Median (IQR)	Mean ± S.D. Median (IQR)
Current Age	71.8 ± 6.9	79.9 ± 5.6 **
Age at diagnosis	68.0 ± 8.1	73.4 ± 7.5 *
Ethnicity	Number (%)	
Caucasian	23 (57.5%)	10 (50%)
Other ethnicities	17 (42.5%)	10 (50%)
Years since diagnosis	2.4 (4.3)	4.9 (7.48) *
PSA at diagnosis (ng/mL)	9.0 (8.2)	132.9 (408.7) **
Gleason Score	Number (%)	
6–7	26 (65%)	6 (30%) *
8–9	14 (35%)	12 (60%)
No histological confirmation	0	2 (10%)
TNM Staging	Number (%)	
T1	9 (23.1%)	0 **
T2	14 (35.9%)	4 (23.5%)
T3	15 (38.5%)	5 (29.4%)
T4	1 (2.6%)	8 (47.1%)
N1	4 (11.1%)	10 (55.6%) **
M1	3 (9.1%)	12 (66.7%) **
ADT duration (years)	-	3.2 (2.4)
WHO performance status	0 (1.0)	1.0 (1.0)
Body weight ^#^	84.5 ± 15.4	86.0 ± 17.1
Body Mass Index BMI ^#^	26.5 ± 4.7	27.7 ± 4.2
Other co-morbidities	Number (%)	
Diabetes	6 (15%)	5 (20%)
Hypertension	22 (55%)	13 (65%)
Ischemic heart disease	10 (25%)	4 (20%)
Smoking	Number (%)	
Past	10 (25%)	8 (40%)
Current	2 (5%)	3 (15%)
Alcohol	Number (%)	
Past	12 (40%)	8 (40%)
Current	11 (28%)	8 (40%)

^#^ Not all participants provided these data; PSA = prostate specific antigen. * Significantly different from LHRHa-naïve participants, *p* < 0.05; ** *p* < 0.001.

**Table 2:** Prevalence data for study cohort—The accepted abnormal values for each parameter are highlighted in bold italics.

Parameter Score	Total Participants (n = 60)	ADT Naïve (n = 40)	ADT (LHRHa) (n = 20)
Total ESS score Number (%)			
≤10	50 (83.3%)	34 (85%)	16 (80%)
>10	10 (16.7%)	6 (15%)	4 (20%)
Global PSQI score Number (%)			
≤5	30 (50%)	24 (60%)	6 (30%) *
>5	30 (50%)	16 (40%)	14 (70%)
Actigraphy parameters Number (%)			
Sleep Efficiency (SE)			
≥80%	37 (61.6%)	23 (57.5%)	14 (70%)
<80%	23 (38.4%)	17 (42.5%)	6 (30%)
Fragmentation Index (FI)			
≤40	25 (41.6%)	21 (52.5%)	4 (20%) *
>40	35 (58.4%)	19 (47.5%)	16 (80%)
Actual Sleep Time (AST)			
≥7 h	34 (56.6%)	20 (50%)	14 (70%)
<7 h	26 (43.4%)	20 (50%)	6 (30%)
Daytime Napping duration			
≤60 min	34 (56.6%)	26 (65%)	8 (40%)
>60 min	26 (43.4%)	14 (35%)	12 (60%)

ESS = Epworth Sleepiness scale; PSQI = Pittsburgh Sleep Quality Index. * Significantly different proportion from LHRH-naïve participants, *p* < 0.05.

**Table 3:** Subjective and objective sleep characteristics of participants who were LHRH naïve or had been on LHRH treatment. PSQI data are presented as number of participants and percentage. ESS data are presented as either mean ± standard deviation or median (interquartile range), depending on data normality.

Total Participants (n = 60)	ADT Naïve (n = 40)	ADT (LHRHa) (n = 20)
PSQI Scoring		
Subjective Sleep quality	Number (%)	
Very good	10 (25%)	2 (10%) *
Fairly good	28 (70%)	12 (60%)
Fairly bad	2 (5%)	5 (25%)
Very bad	0	1 (5%)
Sleep latency score	Number (%)	
0	16 (40%)	2 (10%) **
1–2	16 (40%)	6 (30%)
3–4	2 (5%)	8 (40%)
5–6	6 (15%)	4 (20%)
Sleep duration	Number (%)	
>7 h	16 (40%)	5 (25%)
6–7 h	15 (37.5%)	11 (55)
5–6 h	6 (15%)	3 (15%)
<5 h	3 (7.5%)	1 (5%)
Habitual sleep efficiency	Number (%)	
>85%	21 (52.5%)	2 (10%) **
75–84%	7 (17.5%)	10 (50%)
65–74%	7 (17.5%)	3 (15%)
<65%	5 (12.5%)	5 (25%)
Sleep disturbance score	Number (%)	
0	5 (12.5%)	0
1–9	28 (70%)	13 (65%)
10–18	7 (17.5%)	7 (35%)
19–27	-	-
Use of sleep medication	Number (%)	
Not during the past month	39 (97.5%)	20 (100%)
Less than once a week	-	-
Once or twice a week	-	-
Three or more times a week	1 (2.5%)	0
Daytime functioning score	Number (%)	
0	31 (77.5%)	16 (80%)
1–2	7 (17.5%)	2 (10%)
3–4	1 (2.5%)	2 (10%)
5–6	1 (2.5%)	0
Global PSQI score	4 (4)	7 (3.8) **
ESS Scoring Median (IQR)		
Total ESS Score	5 (6)	8 (5.8)

* Significantly different from ADT-naïve participants, * *p* < 0.05; ** *p* < 0.01.

**Table 4:** Objective sleep characteristics of participants who were LHRH naïve versus those on ADT-LHRHa. Actigraphy data are presented as either mean ± standard deviation or median (interquartile range), depending on data normality.

Selected Actigraphy Parameters		
Total Participants (n = 60)	ADT Naïve (n = 40)	ADT (LHRHa) (n = 20)
Parameters	Median (IQR)	
Actual Sleep Time (h)	6.8 ± 1.3	7.7 ± 1.4 *
Sleep efficiency (%)	81.5 (14.3)	81.4 (7.4)
Wake-bout frequency	39.2 (15.7)	46.2 (15.9) *
Wake-bout duration (min)	100.5 (64.0)	114.5 (34.8)
Fragmentation index	37.4 (23.2)	48.3 (17.9) **
Nap duration (min)	45.2 (43.0)	64.1 (74.5) *
Nap frequency	4.4 (5.3)	7.6 (6.9) *

ESS = Epworth Sleepiness scale; PSQI = Pittsburgh Sleep Quality Index. * Significantly different from ADT-naïve participants, * *p* < 0.05; ** *p* < 0.01.

**Table 5:** The association of androgen deprivation treatment and actigraphic sleep measurements, after adjusting for age, ethnicity, WHO performance score and T cancer staging.

Actigraphic Variables	B	SE	β	*p* Value	R^2^	F (5,50)	Model *p* Value
Total sleep time	0.695	0.520	0.235	0.187	0.088	0.966	0.447
Sleep efficiency	0.388	3.863	0.018	0.920	0.034	0.357	0.875
Fragmentation index	10.626	5.838	0.303	0.075	0.184	2.260	0.063
Nap duration	27.283	16.627	0.282	0.107	0.128	1.466	0.218
Nap frequency	2.709	1.600	0.287	0.097	0.152	1.787	0.133
Sleep latency	−1.105	6.154	−0.032	0.858	0.074	0.798	0.556
Wake-bout frequency	11.629	4.893	0.385	0.021	0.225	2.904	0.022
Wake-bout duration	4.542	15.260	0.054	0.298	0.037	0.379	0.861

## Data Availability

Data are not publicly accessible but can be made available on request to S.M. (Stephen Mangar).
